# Intraoperative Indocyanine Green Retention Test of Left Hemiliver in Decision-Making for Patients With Hepatocellular Carcinoma Undergoing Right Hepatectomy

**DOI:** 10.3389/fsurg.2021.709017

**Published:** 2021-09-17

**Authors:** Tingdong Yu, Xinping Ye, Zhang Wen, Guangzhi Zhu, Hao Su, Chuangye Han, Ketuan Huang, Wei Qin, Xiwen Liao, Chengkun Yang, Zhen Liu, Xiangkun Wang, Banghao Xu, Ming Su, Zili Lv, Wan Yee Lau, Tao Peng

**Affiliations:** ^1^Department of Hepatobiliary Surgery, the First Affiliated Hospital of Guangxi Medical University, Nanning, China; ^2^Department of Hepatobiliary Surgery, the Third Affiliated Hospital of Kunming Medical University, Kunming, China; ^3^Department of Pathology, the First Affiliated Hospital of Guangxi Medical University, Nanning, China; ^4^Faculty of Medicine, The Chinese University of Hong Kong, Hong Kong, China

**Keywords:** hepatocellular carcinoma, indocyanine green retention test, hepatectomy, ALPPS, portal vein ligation, future liver remnant, liver function

## Abstract

**Introduction:** The aim of this study was to select qualified patients with hepatocellular carcinoma (HCC) who underwent right hepatectomy (RH) *via* intraoperative indocyanine green retention test at 15 min (ICG-R15) of the left hemiliver, which prevents severe posthepatectomy liver failure (PHLF).

**Methods:** Twenty HCC patients who were preoperatively planned to undergo RH were enrolled. Intraoperative ICG-R15 of left hemiliver was measured after the right Glissonean pedicle was completely blocked. Patients then underwent RH if intraoperative ICG-R15 was ≤ 10%. Otherwise, patients underwent staged RH (SRH), either associating liver partitioning and portal vein ligation for staged hepatectomy (ALPPS) or portal vein ligation (PVL), followed by stage-2 RH. The comparison group consisted of patients with a ratio of standard left liver volume (SLLV) of > 40% and preoperative ICG-R15 ≤ 10% who underwent RH. The clinical outcomes of these two groups were compared.

**Results:** Of the 20 patients, six underwent stage-1 RH, six underwent ALPPS, five underwent PVL followed by stage-2 RH, and three failed to proceed to stage-2 RH after PVL. No significant differences were found among the 17 patients who underwent stage-1 or stage-2 RH in the study group, the 19 patients in the comparison group, the 11 patients in the stage-2 RH group, and the six patients in the stage-1 RH group in incidences of PHLF, postoperative complications, hospital stay, and HCC recurrence within 1 year after RH. Compared with the stage-1 ALPPS group, the mean operative time and blood loss of the stage-1 PVL group were significantly less (*p* <0.001 and *p* = 0.022, respectively). The stage-1 PVL group had a significantly longer waiting-time (43.4 vs. 14.0 days, *p* = 0.016) than the stage-1 ALPPS group to proceed to stage-2 RH. After stage-2 RH, tumor recurrence within 1 year was 20% (1/5) in patients after PVL and 50% (3/6) after stage-1 ALPPS.

**Conclusions:** Intraoperative ICG-R15 ≤ 10% of left hemiliver was valuable in intraoperative decision-making for patients who were planned to undergo RH. There is a possibility that stage-1 PVL might help to select patients with more favorable biological behavior to undergo stage-2 RH.

## Introduction

Hepatic resection is still the first line treatment for patients with hepatocellular carcinoma (HCC) aiming at cure (1). However, posthepatectomy liver failure (PHLF) and its associated complications remain common after major hepatectomy (2). Right hepatectomy (RH) is a commonly-used surgical procedure for patients with HCC in the right hemiliver. PHLF remains the main cause of hepatectomy-related mortality after RH, especially in patients with a cirrhotic liver (3–5). To accomplish RH for an originally unresectable tumor because of inadequate volume of left hemiliver and to decrease PHLF, portal vein embolization (PVE), portal vein ligation (PVL), and associating liver partitioning and portal vein ligation for staged hepatectomy (ALPPS) are possible surgical strategies to induce remnant left hemiliver hypertrophy (6–8).

The occurrence of PHLF is closely-related to the volume and function of the future remnant left hemiliver (4). Three-dimensional (3D) modeling of the liver based on computed tomography (CT) images can provide accurate remnant liver volume measurement (9, 10). Indocyanine green retention test at 15 min (ICG-R15) is a commonly used and a noninvasive assessment of liver function, which has good predictive values for PHLF (11). Indeed, preoperative ICG-R15 tests have been used to select patients for major hepatectomy (12, 13). While preoperative 3D measurement calculates the only remnant liver volume that is planned to be left behind after liver resection, preoperative ICG-R15 test measures all the functioning non-tumorous parts of the liver, including parts of the liver that are planned to be resected. This study aimed to determine using intraoperative ICG-R15 retention tests to assess only the left hemiliver function in patients who were planned to undergo RH by blocking the right hepatic blood flow. Based on the results of intraoperative ICG-R15 tests, either a stage-1 or stage-2 RH was carried out.

## Patients and Methods

### Patients

Between 2015 and 2018, consecutive patients with hepatitis B virus (HBV)-related HCC in the right hemiliver, who were considered for RH at The First Affiliated Hospital of Guangxi Medical University (Guangxi, China), entered into this study. These patients routinely underwent 64-slice spiral contrast-enhanced CT scanning. Three dimensional (3D) images of the liver and tumor were then reconstructed, and the standard left liver volume (SLLV) was calculated. Informed consent was obtained for these patients to undergo an intraoperative ICG-R15 retention test after blocking the right hepatic blood inflow to select the surgical procedure: either stage-1 or stage-2 RH. When the test was > 10%, the choice of a stage-2 RH using either stage-1 ALPPS or PVL was determined preoperatively by the patients after discussion with the surgical group. A comparison group of patients who underwent RH during the study period was collected from the hospital database. All these patients had a > 40% ratio of SLLV and preoperative ICG-R15 ≤ 10%, but no intraoperative indocyanine green (ICG) retention tests. Based on the Chinese Guidelines (14), Child-Pugh grade A and ICG-R15 < 20–30% are generally considered as the prerequisites for any hepatectomy. A future remnant liver (FRL) volume of more than 40% (for patients with a liver background of chronic hepatitis/cirrhosis) or more than 30% (for patients without that liver background) of the standard remnant liver volume is the prerequisite for major liver resection. Therefore RH is routinely carried out in our unit for HBV-related HCC patients with > 40% ratio of SLLV and a preoperative ICG-R15 ≤ 10%.

The clinicopathologic and perioperative data were collected from the hospital database and pathologic reports. All patients had HBV infection. They all had received antihepatitis viral treatment before and after operations. The tumors were staged according to the Barcelona Clinic Liver Cancer (BCLC) staging system (15). Liver fibrosis and necroinflammatory scores of the non-tumorous tissues were evaluated postoperatively using the Ishak scoring system (16). PHLF was defined according to the International Study Group of Liver Surgery on or after postoperative day 5 using the increased international normalized ratio and hyperbilirubinemia, as defined by the normal cut-off levels of the local laboratory (17). Postoperative complications were evaluated using the Clavien–Dindo classification (18). This study was conducted with approval from the Ethics Committee of the First Affiliated Hospital of Guangxi Medical University. Patients gave informed consent for the operations and for their data to be used for clinical research.

### Analysis of 3D Reconstruction and Left Liver Volume Measurements

Computed tomography images, including non-enhanced and three-phase contrast-enhanced scans (arterial, portal venous, and delayed phases), were obtained from a 64-slice spiral CT scanner. The CT scanning data was inputted in IQQA-Liver (EDDA Technology, Princeton, NJ, USA). The spatial structures including the tumor, hepatic artery, portal vein, and liver, were then reconstructed as a 3D model and the actual total liver volume (TLV) and left liver volume (LLV) were automatically calculated. The SLLV was calculated according to the following formula to estimate the liver volumes of the Chinese patients: standard liver volume (SLV [cm^3^]) = 11.508 × body weight (kg) + 334.024 and SLLV = SLV × LLV/TLV (19).

### Intraoperative ICG Retention Test and Selection of Surgical Procedures

Indocyanine green retention test at 15 min was measured using a nasal mucosa probe of Hitachi 7700 (Tokyo, Japan) by injecting a dose of 0.5 mg/kg of ICG rapidly *via* a peripheral vein of the forearm when the right Glissonean pedicle at the hepatic hilus was completely blocked for 5 min intraoperatively. An ICG-R15 ≤ 10% of the left remnant liver was considered adequate to proceed to a stage-1 RH. In contrast, patients were planned to undergo a stage-2 RH when the ICG-R15 was > 10%. The stage-1 operations included ALPPS or PVL following the wish of the patients before surgery.

### Statistical Analysis

Quantitative data were expressed as mean and standard deviation and compared using the Student's *t*-test. Qualitative variables are expressed as numbers and percentages and compared using Fisher's exact tests. Spearman's rank-order correlation was used to evaluate the relationship with PHLF, the SLLV ratio, the necroinflammatory scores, and the fibrosis scores. Statistical analysis was performed using SPSS (version 21.0, IBM), and a *P*-value < 0.05 was considered statistically significant.

## Results

### Patient

As shown in Table 1, 20 patients with HBV-related HCC in the right hemiliver who required RH were included into this study. These patients accepted intraoperative ICG retention tests to select between the stage-1 or stage-2 RH. Specifically, six patients underwent stage-1 RH and 14 patients underwent stage-2 RH. During the same time period, 19 patients who had preoperative ICG-R15 ≤ 10% and adequately determined over 40% ratio of SLLV underwent stage-1 RH without intraoperative ICG-R15 tests. These patients were used as the comparison group. Our data showed that SLLV (445.8 mL) and SLLV ratio of 44.3% in the ICG test group was significantly less compared with the SLLV (558.9 mL) and SLLV ratio (55.0%) in the comparison group (*p* < 0.002 and *p* < 0.001, respectively), and the preoperative ICG-R15[7.0(2.1–11.5)%] in ICG test group was higher compared with ICG-R15 [5.3(0.9–9.8)%] in the comparison group (*p* = 0.045). There were no significant differences between the two groups in age, gender, Child–Pugh grading, tumor number, tumor size, and BCLC staging.

**Table 1:** Demography of HCC Patients who underwent RH.

**Variables**	**Intraoperative ICG-R15 test group** **(*n* = 20)**	**Comparison group** **(*n* = 19)**	***P-*value**
Age, yrs	43.7(35–50)	42.5 (20–69)	0.718
Female/male	2/18	5/14	0.235
Child–Pugh class A/B	19/1	18/1	1.000
**Tumor**
HCC	19	19	1.000
HCC-ICC	1	0	
SLLV, ml	445.8 (262.1–613.4)	558.9(343.6–786.2)	0.002
Ratio of SLLV, %	44.3 (23.0–66.6)	55.0(41.4–73.8)	0.001
Preoperative ICG-R15, %	7.0(2.1–11.5)	5.3(0.9–9.8)	0.045
**Tumor number**, ***n***
1	14	13	1.000
≥2	6	6	
Tumor size, cm	9.2(3.5–17)	11.0(3.0–17)	0.176
**PVTT**, ***n*****(%)**
I	2(10.0%)	2(10.5%)	1.000
II	6(30.0%)	0	NA
III	0	1(5.3%)	NA
MVI	10(62.5%)	11(57.9%)	1.00
Missing	3	0	
BCLC stage A/B	15/5	12/7	0.501

*ICG, indocyanine green; HCC, hepatocellular carcinoma; HCC-ICC, combined hepatocellular carcinoma and intrahepatic cholangiocarcinoma; SLLV, standard left liver volume; PVTT, portal vein tumor thrombus; MVI, microvascular invasion; RH, right hepatectomy*.

### Outcomes of the Intraoperative ICG Retention Test Group and the Comparison Group

**Figure 1:**
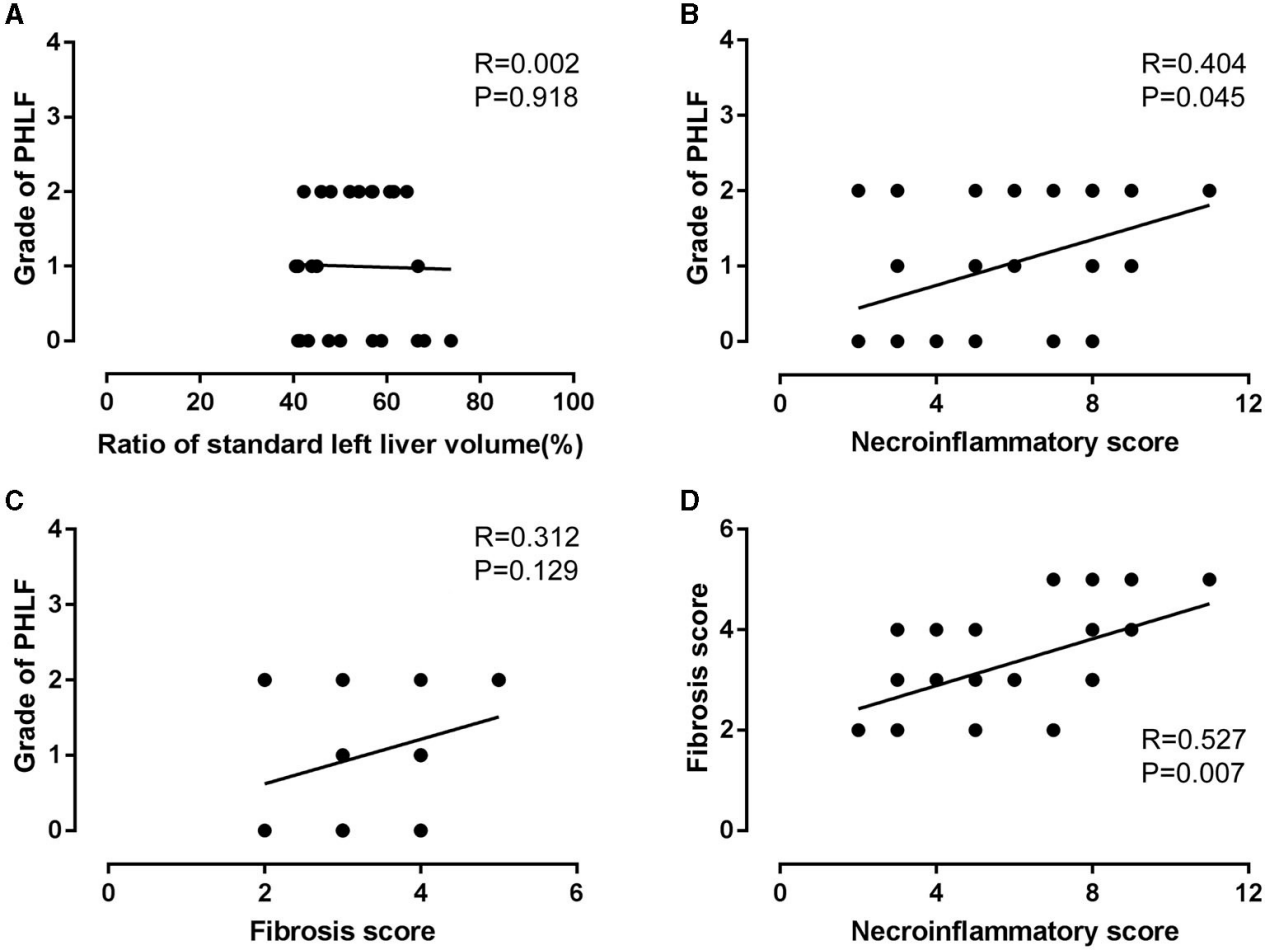
Association analysis of grade of PHLF with ratio of standard left liver volume **(A)**, liver fibrosis score **(B)** and necroinflammatory score **(C)**, fibrosis score with necroinflammatory score **(D)** for total 25 patients with RH.

Based on the results of intraoperative ICG-R15 tests, of the 20 patients in the study group (Table 2), six patients finally underwent stage-1 RH and 14 patients were planned to undergo stage-2 RH using either stage-1 ALPPS (*n* = 6) or stage-1 PVL (*n* = 8). Of these 14 patients who were under stage-1 operations, 11 were able to proceed to stage-2 RH (after stage-1 ALPPS, *n* = 6, after stage-1 PVL, *n* = 5) because of adequate growth in volumes of the FRL, and the remaining three cases failed for stage-2 RH due to tumor progression. The fibrosis stages (S3 + S4) of the livers in the 17 patients with planned stage-2 RH (64.7%, 11/17) were more severe, though insignificant, than the 19 patients in the comparison group (36.8%, 7/19) (*p* > 0.005). However, our results showed that the incidences of PHLF, Clavien–Dindo grading, hospital stay, and recurrence within 1 year after RH in the intraoperative ICG test group were not significantly worse than the comparison group. A combined analysis of all 25 patients who underwent stage-1 RH (six in the intraoperative ICG-R15 group and 19 in the comparison group) suggested that PHLF following RH was significantly associated with the liver necroinflammatory score (*r* = 0.404, *p* = 0.045; Figure 1B), but not with SLLV (Figure 1A) and fibrosis score (Figure 1C). However, the liver fibrosis score was closely related to the liver necroinflammatory score (*r* = 0.527, *p* = 0.007; Figure 1D).

**Table 2:** Outcomes of RH in intraoperative ICG test group and comparison group.

**Variables**	**Intraoperative ICG-R15 test group** **(*n* = 20)**	**Comparison group** **(*n* = 19)**	***P-*value**
**Surgical procedure**, ***n*****(%)**
RH	6(30.0%)	19(100%)	<0.001
Staged procedure	14(70.0%)	0	
**RH completion**, ***n*****(%)**
RH	6(35.3%)	19(100%)	<0.001
SRH	11(64.7%)	0	
Failure for SRH	3		
**Ishak score**
Fibrosis stage, *n* (%)			0.181
Stage 1+ stage 2	6(35.3%)	12(63.2%)	
Stage 3+ stage 4	11(64.7%)	7(36.8%)	
Necroinflammatory grade, *n* (%)			0.981
Grade 1	4(23.5%)	4(21.0%)	
Grade 2	8(47.1%)	9(47.4%)	
Grade 3	5(29.4%)	6(31.6%)	
PHLF, (*n*, %)			0.505
None	10(58.8%)	8(42.1%)	
Grade A+B	7(41.2%)	11(67.9%)	
Clavien-Dindo, *n* (%)			0.281
Grade 1	10(58.8%)	15(78.9%)	
Grade 2	7(41.2%)	4(21.1%)	
Hospital stay after surgery, days	14(7–47)	10(6–22)	0.122
Recurrence within 1 year, *n* (%)	6(35.3%)	7(36.8%)	1.000
missing	0	1	

*RH, right hepatectomy; SRH, staged right hepatectomy; PHLF, posthepatectomy liver failure*.

### Analysis of Stage-2 RH With Intraoperative ICG Retention Tests

Comparing the 11 patients who underwent stage-2 RH with the six patients who underwent stage-1 RH based on the intraoperative ICG-R15 tests, no significant differences were found in SLLV and in SLLV ratio. However, the preoperative ICG-R15 and intraoperative ICG-R15 in patients who underwent stage-2 RH were significantly higher than those patients who underwent stage-1 RH (*p* = 0.01 and *p* < 0.001, respectively, Table 3). After the left hemilivers had increased to adequate volumes in the 11 patients who underwent stage-2 RH, the surgical time, blood loss, PHLF grading, postoperative complications, and hospital stay were similar to the six patients who underwent stage-1 RH, with the incidence of PHLF in the stage-2 RH group (27.3%, 3/11) being less than the stage-1 RH group (66.7%, 4/6).

**Table 3:** Outcomes of SRH and RH with intraoperative ICG-R15 test in the study group.

**Variables**	**SRH (*n* = 11)**	**RH (*n* = 6)**	***P-*value**
Intraoperative ICG-R15, %	14.8 (10.4–24.1)	6.6 (2.6–9.2)	<0.001
Preoperative ICG-R15, %	7.4 (3.7–11.5)	4.1 (2.1–6.3)	0.01
SLLV, ml	428.6 (262.1–568.1)	462.6 (349.3–613.4)	0.538
SLLV ratio, %	42.3 (23.0–52.5)	47.2 (41.0–66.6)	0.292
SLLV before RH, ml	573.2 (450.1–758.2)	462.6 (349.3–613.4)	0.027
SLLV ratio before RH, %	56.2 (40.0–67.9)	47.2 (41.0–66.6)	0.071
Operation time, minutes	300.7 (213–515)	372.5 (217–448)	0.234
Blood loss, ml	981.8 (300–2,500)	908 (350–1,600)	0.815
**Ishak score**
Fibrosis stage, *n* (%)			0.600
Stage 1+ stage 2	3(27.3%)	3(50.0%)	
Stage 3+ stage 4	8(72.7%)	3(50.0%)	
Necroinflammatory grade, *n* (%)			0.090
Grade 1	1(9.1%)	3(50.0%)	
Grade 2	7(63.6%)	1(16.7%)	
Grade 3	3(27.3%)	2(33.3%)	
PHLF, *n*(%)			0.162
None	8(72.7%)	2(33.3%)	
Grade A+B	3(27.3%)	4(66.7%)	
Clavien-Dindo, *n*(%)			1.000
Grade 1	6(54.5%)	4(66.7%)	
Grade 2	5(45.5%)	2(33.3%)	
Hospital stay after surgery, days	15(8–47)	12(7–27)	0.598
Recurrence within 1 year, *n* (%)	3(27.3%)	3(50.0%)	0.600
missing	0	0	

*RH, right hepatectomy; SRH, staged right hepatectomy; PHLF, posthepatectomy liver failure*.

### Comparison of Stage-1 Surgery Between the ALPPS and PVL Groups

The data of the six patients who underwent stage-1 ALPPS and the eight patients who underwent stage-1 PVL are shown in Table 4. In the PVL group, five patients underwent laparoscopic stage-1 operation. The average operative time and blood loss in the PVL group were significantly less compared with the stage-1 ALPPS group (*p* < 0.001 and *p* = 0.022, respectively). Two patients developed PHLF and one had bile leakage after the stage-1 procedure in the ALPPS group, whereas three patients in the PVL group failed to proceed to the stage-2 RH due to tumor progression and inadequate growth in volumes of FRL.

**Table 4:** Comparison of staged-1 clinical characteristics of SRH between the ALPPS group and PVL group.

**Variables**	**ALPPS (*N* = 6)**	**PVL group (*N* = 8)**	***P*-value**
SLLV, ml	484.9 (396.5–568.1)	408.3 (262.1–649.7)	0.157
Ratio of SLLV, %	47.1 (41.0–52.5)	40.0 (23.0–57.6)	0.122
IntraoperativeICG-R15, %	13.4 (12.0–15.8)	17.0 (10.4–24.1)	0.081
Laparoscopic surgery	0	5	NA
Operative time, minutes	349.5 (293–408)	164.6 (92–229)	<0.001
Blood loss, ml	433.3 (200–600)	185.0 (20–500)	0.022
PHLF, *n* (%)	2(33.3%)	0	NA
Bile leak, *n* (%)	1(16.7%)	0	NA
Failure for Stage 2, n (%)	0	3(37.5%)	NA

*ALPPS, associating liver partition and portal vein ligation for staged hepatectomy; PVL, portal vein ligation*.

### Comparison of Stage-2 RH Between the ALPPS and PVL Groups

All six patients in the stage-1 ALPPS group and five in the stage-1 PVL group completed stage-2 RH. Two patients underwent transarterial chemoembolization (TACE) after the stage-1 PVL because of a long wait for liver hypertrophy to reach adequate volumes in the FRL. Our results demonstrated that patients in the PVL group had to wait for a significantly longer time (43.4 days) to undergo the stage-2 RH when compared with the ALPPS group (14.0 days) (*p* = 0.016, Table 5). There were no significant differences in increased volume per day, increased standard volume per day, operative time, blood loss, fibrosis scoring, Clavien–Dindo scoring after the operation, postoperative hospital stay, and HCC recurrence within 1 year between the two groups (Table 5).

**Table 5:** Comparison of staged-2 clinical characteristics of SRH between the ALPPS group and PVL group.

**Variables**	**ALPPS** **(*N* = 6)**	**PVL group (*N* = 5)**	***P-*value**
TACE after stage 1	0	2	NA
Time to second stage, days	14.6(12–22)	44.2(21–83)	0.016
Increased in volume per day, ml	12.7 (5.2–14.9)	9.8(1.2–18.6)	0.522
Ratio of gained standard volume per day, %	1.2(0.5–1.5)	1.0(0.1–1.8)	0.536
Operative time of stage 2, minutes	338.6(140–515)	255.2(185–450)	0.274
Blood loss, ml	1016.7 (300–2,500)	940.0 (350–1,500)	0.855
PHLF, *n*(%)	1(16.7%)	2(40%)	0.545
**Clavien-Dindo grade**
Grade 1/2	3/3	3/2	1.000
Bile leak, *n*(%)	3(50%)	3(60%)	1.000
Hospital stay after surgery, days	11(8-19)	20(8–47)	0.230
Recurrence within 1 year, *n* (%)	3(50%)	1(20%)	0.545

### Clinical Outcomes of the Two Patients With TACE After PVL

**Figure 2:**
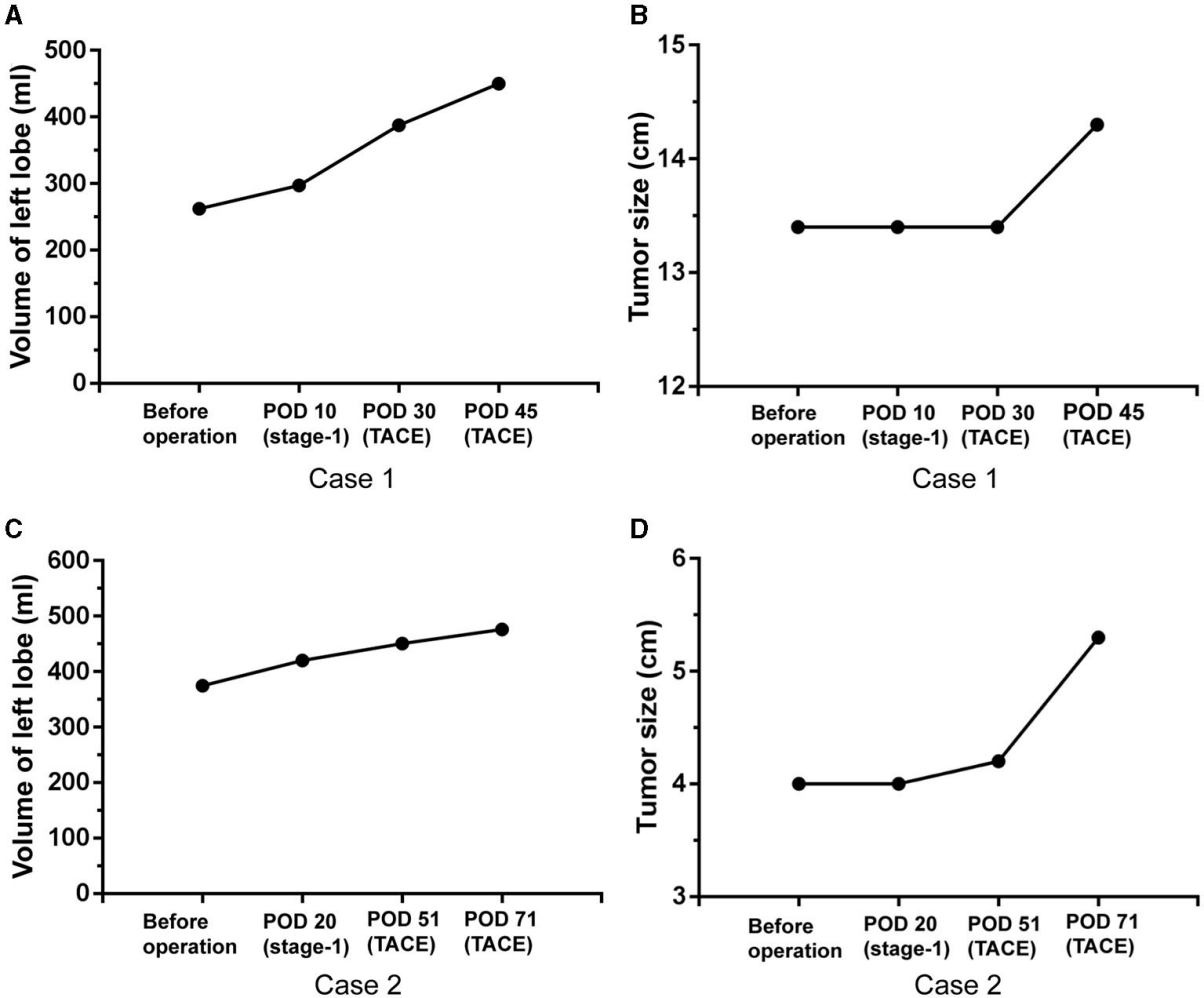
Volume increase and tumor progression in two patients with TACE after PVL. Volume increase of left lobe **(A)** and tumor size change **(B)** after PVL and TACE in the first case, volume increase of left lobe **(C)** and tumor size change **(D)** after PVL and TACE in the second case. POD: postoperative day.

Two patients underwent two sessions of TACE after PVL to slow down tumor progression for the slow increase in volumes of remnant left hemiliver. Finally, the increased volume and SLLV ratio in one patient increased from 262.1 mL (23.0%) to 450.1 ml (39.5%) and another patient increased from 374.8 ml (37.0%) to 476.1 ml (47.0%; Figures 2A,C). These two patients underwent stage-2 RH although the tumor sizes had increased slightly from 13.4 cm to 14.3 cm, and from 4.0 cm to 5.3 cm, respectively (Figures 2B,D).

## Discussion

Right hepatectomy is a major hepatectomy. In general, the accepted remnant left hemiliver volume for safe liver resection ranges from 20–30% in patients without any underlying liver diseases (4, 20–22). However, in patients with chronic hepatitis/cirrhosis, the recommended critical minimum ratio of left hemiliver volume is 40% to support postoperative liver function (22). Thus, precise measurement of the left hemiliver volume before hepatectomy is helpful and necessary. Three-dimensional CT reconstruction provides a good method to calculate left hemiliver volume (10). However, accurate volumetrics do not necessarily represent an adequate liver function in the remnant liver because patients with chronic hepatitis/cirrhosis have impaired liver functions. Thus, the risk associated with PHLF in hepatectomy cannot solely rely on precise volume measurement of the remnant liver, but also on the adequate assessment of effective hepatic function. It is well-known that ICG-R15 (11, 13) and technetium-99m (Tc-99m) mebrofenin (23), a medical radioactive isotope, were commonly used for accurate assessment of liver function and risk prediction of PHLF occurrence. A clinical study presented that Tc-99m mebrofenin blood clearance rate at 15 min was significantly correlated with the 15-min ICG clearance rate (24). ICG-R15 was known to mainly measure the global liver function, but Tc-99m mebrofenin scintigraphy could calculate not only the global liver function but also the effective FRL function (25), which is probably helpful to assess the left hemiliver function and to select qualified patients for RH. However, due to the unavailability of Tc-99m mebrofenin in our hospital, intraoperative ICG-R15 of the left liver was conducted to help operative decision-making in selecting patients for stage-1 or stage-2 RH.

The Institutional Review Board of our hospital only allows surgeons to carry out RH when the preoperative ICG-R15 test is ≤ 10% and the SLLV ratio is over 40% in patients with a background of chronic hepatitis, as this puts the patients to be at low risks of PHLF. However, the preoperative ICG-R15 tests assess the function of the whole liver, including parts of the liver which are going to be resected in RH. It is therefore the design of this study to carry out intraoperative ICG-R15 in patients who are planned to undergo RH. In this study, there were two groups of patients who were planned to undergo RH, namely, the intraoperative ICG test group and the comparison group. The data from the former group was collected prospectively whereas those from the latter group were collected retrospectively. When the two groups were compared, the former group had a significantly (*p* = 0.001) lower ratio of SLLV% (44.3 ± 8.8%) than the latter group (55.0 ± 9.6%), and the preoperative ICG-R15% was significantly higher than the latter group [7.0(2.1–11.5)% vs. 5.3(0.9–9.8)%, *p* = 0.045]. The hypothesis of this study is that the additional intraoperative ICG test of only the left hemiliver function would help to discriminate those who are at high risks of developing PHLF after RH (left-liver ICG-R15 > 10%). Table 2 shows that intraoperative ICG-R15 helped to identify patients with adequate liver function to undergo RH. Only Grade A and B PHLF developed in those patients with incidences that were similar to those patients who were assessed preoperatively to have adequate volumes and functions to undergo RH. This study also showed that intraoperative ICG-R15 is more accurate in assessing the function of the liver remnant as only the function of the left hemiliver was assessed intraoperatively, whereas all the non-tumorous parts of the whole liver were assessed by preoperative ICG-R15.

The preoperative ICG clearance test is commonly used and it has a high predictive value in assessing hepatic functional reserve, particularly in patients undergoing major hepatectomy (13). The future liver remnant plasma clearance rate of ICG (preoperative plasma clearance rate of indocyanine green × % FRL) has been found useful in predicting post hepatectomy liver function (26, 27). In this study, the ICG-R15 clearance test was conducted after blocking the right hepatic blood flow to help decision-making for stage-1 or stage-2 RH in 20 patients who preoperatively planned to undergo RH. Based on a cut-off value of intraoperative ICG-R15 of ≤ 10%, six patients underwent stage-1 RH and 14 were planned to undergo stage-2 RH. Finally, 17 patients underwent RH, including six for stage-1 RH and 11 for stage-2 RH. Although the degrees of fibrosis were more severe in the intraoperative ICG retention test group, clinical outcomes including incidences of PHLF, Clavien–Dindo classification of complications, hospital stay, and HCC recurrence within 1 year after stage-1 RH and stage-2 RH were similar. Using the cut-off value of ≤ 10% for intraoperative ICG-R15, 11 patients underwent stage-2 RH whereas six underwent stage-1 RH. There were no significant differences in SLLV and SLLV ratio between these two groups. However, the preoperative ICG-R15 and intraoperative ICG-R15 in those patients who underwent stage-2 RH were significantly different from those patients who underwent stage-1 RH (*p* = 0.01 and *P* < 0.001, respectively). All the 11 patients underwent stage-2 RH after adequate increase in volumes of remnant left hemiliver. The incidence of PHLF was lower than stage-1 RH. These results showed that “both volume and function” are important, and intraoperative ICG retention test is helpful in intraoperative decision-making for either stage-1 or stage-2 RH for patients who are considered to undergo RH to avoid severe PHLF and posthepatectomy complications. Our data also revealed that PHLF after RH was closely related to the necroinflammatory scoring in patients with intraoperative ICG-R15 ≤ 10% of left hemiliver and SLLV ≥ 40% (*r* = 0.404, *p* = 0.045). The liver fibrosis score was correlated with the necroinflammatory score (*r* = 0.527, *p* = 0.007).

Our results demonstrated that the operative time and blood loss during stage-1 ALPPS were significantly more than in stage-1 PVL (*p* < 0.001 and *p* = 0.022, respectively). Furthermore, two patients (33.3%) suffered from PHLF and one patient (16.7%) developed bile leakage after stage-1 ALPPS. There was also no complication in the stage-1 PVL, indicating that PVL is safer. However, patients in the ALPPS group spent significantly shorter waiting time and more patients were able to proceed to stage-2 RH compared with the PVL group, suggesting that ALPPS was a better choice for completion of the stage-2 RH than PVL. The price to pay for stage-1 ALPPS was a higher probability of PHLF and bile leakage. These results were consistent with the previously reported study that ALPPS had a significantly higher rate of complete liver resection in patients with initially unresectable liver tumors with inadequate volumes of the remnant liver when compared with PVE/PVL, but at the cost of higher complication and mortality rates (28).

In this small-sample-size study, although patients in the PVL group had to wait for a significantly longer time (43.4 days) for stage-2 RH compared with the ALPPS group (14.0 days), PVL had the advantages of less operative time and less blood loss. Three patients failed to reach to stage-2 RH due to tumor progression on days-4, 15, 21, respectively after stage-1 PVL. There was a significantly lower tumor recurrence rate within 1 year in the stage-1 PVL group (20%, 1/5) when compared with the stage-1 ALPPS group (50%, 3/6). However, whether this stage-1 PVL can be used as a “wait and see” strategy to discriminate HCC patients with more biologically favorable HCC to undergo stage-2 RH requires large-sample-sized studies to confirm.

A previously reported study showed that rescue TACE was helpful to increase the waiting time for the future liver remnant to grow adequately in size for stage-2 hepatectomy after laparoscopic-associated liver tourniquet and portal ligation (29). In our study, two patients underwent TACE after stage-1 PVL, and the procedure was effective in limiting tumor progression to wait for the remnant left hemiliver to increase adequately in volume. Finally, these two patients successfully completed stage-2 RH. Regrettably, one had a tumor recurrence within a short period of time (7 months) after stage-2 RH.

In conclusion, our results suggested that intraoperative ICG-R15 is helpful in the decision-making of stage-1 or stage-2 RH for HCC patients. An intraoperative ICG-R15 ≤ 10% of left hemiliver was proposed to be the good cut-off value for safe RH. However, the present study has the following limitations. First, the sample size of the study is small, future large-sample-size studies are needed to verify the findings of this study. Second, although part of the study was prospective (the intraoperative ICG-R15 study group), part of it was retrospective (the comparison group). There are potential inherent biases in such a type of study. Third, all the patients in this study were patients with HBV-related HCC. The results of this study may not be extrapolated to patients with other etiologies of HCC. Finally, this study was conducted on patients with HCC who were planning to undergo RH. The results may not be applicable to other types of liver resection.

## Data Availability Statement

The original contributions presented in the study are included in the article/supplementary material, further inquiries can be directed to the corresponding author/s.

## Ethics Statement

The studies involving human participants were reviewed and approved by the Ethics Committee of the First Affiliated Hospital of Guangxi Medical University. The patients/participants provided their written informed consent to participate in this study.

## Author Contributions

TP designed the study and revised the manuscript. TY analyzed the data and wrote the manuscript. XY codesigned the study and performed an intraoperative indocyanine green retention test. WL revised the manuscript and language editing. ZW, GZ, HS, and BX provided study patients and carried out the intraoperative indocyanine green retention test. CH, KH, and WQ performed the liver volume measurement and checked the data. XL, CY, ZL, and XW performed preoperative indocyanine green retention test and clinical data collection. ZL completed pathological diagnosis and MVI assessment. All authors contributed to the article and approved the submitted version.

## Funding

This work was supported in part by the National Nature Science Foundation of China (No. 81560535, 81072321, 30760243, 30460143, and 30560133), 2009 Program for New Century Excellent Talents in University (NCET), Guangxi Nature Sciences Foundation (No. GuiKeGong 1104003A-7), and Guangxi Health Ministry Medicine Grant (Key-Scientific Research-Grant Z201018).

## Conflict of Interest

The authors declare that the research was conducted in the absence of any commercial or financial relationships that could be construed as a potential conflict of interest.

## Publisher's Note

All claims expressed in this article are solely those of the authors and do not necessarily represent those of their affiliated organizations, or those of the publisher, the editors and the reviewers. Any product that may be evaluated in this article, or claim that may be made by its manufacturer, is not guaranteed or endorsed by the publisher.
